# Application of Nipple Aspirate Fluid miRNA Profiles for Early Breast Cancer Detection and Management

**DOI:** 10.3390/ijms20225814

**Published:** 2019-11-19

**Authors:** Cathy B Moelans, Susana I. S. Patuleia, Carla H. van Gils, Elsken van der Wall, Paul J. van Diest

**Affiliations:** 1 Department of Pathology, University Medical Center Utrecht—Utrecht University, 3508 GA Utrecht, The Netherlands; S.I.SchoutenPatuleia-7@umcutrecht.nl (S.I.S.P.); P.J.vanDiest@umcutrecht.nl (P.J.D.); 2 Department of Epidemiology of the Julius Center for Health Sciences and Primary Care, University Medical Center Utrecht—Utrecht University, 3508 GA Utrecht, The Netherlands; C.vanGils@umcutrecht.nl; 3 Department of Medical Oncology, University Medical Center Utrecht—Utrecht University, 3508 GA Utrecht, The Netherlands; E.vanderWall@umcutrecht.nl

## 1. Commentary

The authors of the recently published review, “Why the Gold Standard Approach by Mammography Demands Extension by Multiomics? Application of Liquid Biopsy miRNA Profiles to Breast Cancer Disease Management”, elegantly describe miRNAs as potential blood-based biomarkers for early breast cancer detection [1]. However, remarkably, they fail to mention the potential of nipple aspirate fluid (NAF) as a source of miRNAs. The best liquid biopsies for developing a screening diagnostic tool are those readily accessible and in close proximity to the disease area, such as NAF for breast cancer. NAF is secreted in small amounts by the breast ducts of adult non-lactating women, and can be collected by non-invasive vacuum aspiration. The use of oxytocin nasal spray promotes the release of already existing fluid in the milk ducts, thereby yielding sufficient material for molecular analysis in the majority of healthy volunteers and breast cancer patients [2,3,4,5,6,7]. NAF collection causes significantly less discomfort compared to other breast cancer screening modalities [3,4], and we and others have shown the feasibility of measuring miRNAs in NAF, pathological nipple discharge, and breast ductal fluids [7,8,9,10]. In fact, we have demonstrated that using the same technique and thresholds, but more sample volume input (200 µL serum instead of 20 µL NAF), merely 144/754 (19%) profiled miRNAs could be detected in serum from healthy women, as opposed to 240/754 miRNAs (32%) in NAF [9]. This suggests that NAF contains more miRNAs than serum, and hence, is enriched with these biomarkers. In comparative miRNA analysis between NAF, normal breast tissue, milk, serum and plasma, normal breast tissue was the sample type that shared the highest number of miRNAs with NAF, followed by milk, serum and plasma. This points to a distinct miRNA pattern in NAF, probably best reflecting the breast microenvironment. As the majority of most abundant miRNAs in NAF have established tumor suppressor (e.g., hsa-miR-205-5p and hsa-miR-203a-3p) or oncogenic (e.g., the miR-23a~27a~24-2 cluster) roles, there is potential clinical applicability of miRNA NAF analysis in early detection and management of breast cancer. Studies comparing cancerous and healthy NAF are underway [11]. Hence, although we agree with Zubor et al. that blood-based liquid biopsies have great potential to improve breast cancer screening and management, and acknowledge that blood is a more convenient liquid biopsy, we believe that NAF, derived directly from the breast ductal system, may provide at least additional but potentially more specific and sensitive information.
